# 电磁导航支气管镜矢量定位法在肺小结节手术中的应用

**DOI:** 10.3779/j.issn.1009-3419.2019.11.05

**Published:** 2019-11-20

**Authors:** 桂松 宋, 桐 邱, 云鹏 玄, 艳东 赵, 文捷 矫

**Affiliations:** 266003 青岛，青岛大学附属医院胸外科 Department of Thoracic Surgery, the Affiliated Hospital of Qingdao University, Qingdao 266003, China

**Keywords:** 肺小结节, 电磁导航支气管镜, 矢量定位, 电视胸腔镜手术, Pulmonary nodule, Electromagnetic navigation bronchoscopy, Vectorial localization, Video-assisted thoracic surgery

## Abstract

**背景与目的:**

随着计算机断层扫描技术（computed tomography scans, CT）的广泛应用，越来越多的肺小结节被发现，一些需要外科手术处理的结节数量也在增加。胸腔镜手术中对于不可直视及触摸到的肺外周小结节的准确定位较为困难。虽然目前一些常用的定位技术能够带来比较好的定位效果，如CT引导的穿刺定位和电磁导航支气管镜（electromagnetic navigation bronchoscopy, ENB）系统引导的亚甲兰染色定位，但同时仍存在着气胸、出血及定位不准确的问题。ENB引导的矢量定位法是我中心首创的一种新型定位技术，该技术避免了前两种

**方法:**

可能出现的胸膜损伤或者定位区域扩大的问题，为ENB引导的定位技术提供了一定的指导。本文回顾性分析胸腔镜术前通过ENB引导矢量定位的临床结果来确定该方法的临床应用价值。方法回顾性分析2017年10月-2018年10月于青岛大学附属医院胸外科行ENB矢量定位法进行胸腔镜手术前定位的患者资料，统计患者临床资料、肺小结节影像学特征，评估该方法临床应用的安全性及有效性。

**结果:**

我们成功实施了22例患者的22个肺外周结节在ENB引导下矢量定位和腔镜下楔形切除（22/22, 100%）。结节平均大小为（11.0±3.6）mm，距脏层胸膜表面距离为（16.5±6.2）mm；ENB系统显示屏导航定位装置（locatable guide, LG）与病灶距离为（14.5±10.1）mm，离体标本标记与病灶距离为（15.3±11.0）mm，ENB矢量定位平均时间为（17.5±4.2）min，无定位点LG发生移位（0.0%）。手术过程无中转开胸（0.0%），患者术中及术后未见明显并发症（0.0%），术后平均住院时间为（3.8±1.2）d，无围手术期患者死亡（0.0%）。术后病理结果为恶性肿瘤者19例，均得到了完全切除。

**结论:**

我们应用ENB引导的矢量定位法在肺外周小结节术前定位和微创切除的初步经验提示该方法安全、可行、有效，可作为ENB操作中可选的一种定位方式。胸外科临床医师可以进一步研究该方法并应用到临床操作中。

## Abstract (en)

**Background and Objective:**

More patients with pulmonary nodules are being referred to thoracic surgeons under the increasing use of computed tomography scans (CT). Impalpable peripheral subpleural solitary pulmonary nodules are difficult to be localized by video assisted thoracic surgery. Although some common techniques including CT-guided puncture positioning and electromagnetic navigation bronchoscopy (ENB)-guided methylene blue staining positioning, can bring good Results in positioning, there are still some complications such as pneumothorax, hemorrhage and inaccurate positioning. Vectorial localization guided by electromagnetic navigational bronchoscopy followed by thoracoscopic resection is a novel alternative technique by us firstly for definitive diagnosis, which can avoid the possible injury of pleural or enlargement of the location area, providing some guidance for ENB-guided location technology. The main Objective of this study was to evaluate the feasibility and our initial experience of vectorial localization guided by electromagnetic navigation followed by video-assisted thoracoscopic pulmonary solitary nodules resection.

**Methods:**

We retrospectively analyzed 22 cases who undergoing vectorial localization of peripheral pulmonary lesion guided by electromagnetic navigation prior to video assisted lung resection, and characteristics and intraoperative outcomes were explored.

**Results:**

Twenty-two nodules of twenty-two patients were all localized by this method successfully with an average location time (17.5±4.2) min. The average nodule size was (11.0±3.6) mm. The distance between the locatable guide probe (LG) and lesion on the electromagnetic navigation bronchoscopy screen was (14.5±10.1) mm. The distance between the lesion and probe mark on the dissected specimen was (15.3±11.0) mm. There was no displacement of any case. No conversion to thoracotomy was found. And there were no adverse events during the localization and operation procedure. Length of hospital stay was (3.8±1.2) d and the operative mortality was 0.0%. Malignant lesions were found in 19 patients and they were all completely resected with negative microscopic margins.

**Conclusion:**

Our initial experience with vectorial localization of peripheral pulmonary lesion guided by electromagnetic navigation and minimally invasive resection proved that this technique was an alternative accurate and safe way for small pulmonary nodules. Thoracic surgeons should further investigate this method and apply it to clinical practice.

近年来，在人们日益增长的健康需求和高分辨率多层电子计算机断层扫描技术（high resolution computed tomography, CT）的广泛应用下，肺小结节（small pulmonary nodules, SPNs）的检出也越来越多^[1]^。肺小结节是指影像学上表现为边界清楚、直径≤3 cm、周围被含气肺组织包绕的类圆形或不规则形的病灶，并且不伴有肺不张、肺门增大及胸腔积液等表现^[2]^。由于有一部分结节有恶性的可能，因此需要外科手术切除确诊及治疗的肺小结节也在增加^[3]^。对于电视胸腔镜手术（video-assisted thoracoscopic surgery, VATS）来说，那些直径 < 1 cm或者胸膜下深度超过0.5 cm的病灶，尤其还有磨玻璃结节（ground glass nodules, GGO）难以通过手指触摸定位^[4, 5]^，在术前及术中对该类小结节进行准确定位从而做到精准切除至关重要。

在肺外周结节不同的定位方法中，CT引导下经皮穿刺带钩金属丝（hook wire）定位是一种较为广泛应用的定位方法，虽然该方法比较安全、有效，但穿刺定位后可出现气胸、出血和疼痛等情况^[6, 7]^，甚至hook wire发生移位甚至脱落也时有发生^[8, 9]^。近年来，利用电磁导航支气管镜（electromagnetic navigation bronchoscopy, ENB）系统引导的肺小结节定位是一项新的安全、有效的微创定位技术^[10, 11]^。ENB技术不仅并发症的风险较低，而且相较于CT引导穿刺定位技术而言，后者需要患者在CT室和手术间中转，而ENB技术可以实现在同一手术间、由同一手术团队完成对病灶定位、切除^[12]^。

为了提高ENB引导的定位精度，我们应用了一种不使用染料的新方法——矢量定位法（vectorial localization of peripheral pulmonary lesion guided by ENB）^[13]^，即把导航定位装置（locatable guide, LG）的传感器探头作为定位标志，在胸腔镜手术中保留LG作用于病灶的矢量位置来达到精准切除病灶的目的，具体操作过程如下文所述。本文通过对本中心于2017年10月-2018年10月经过ENB矢量定位法对肺小结节进行术前定位的22例患者的临床效果进行结果报告，以期分析该方法的临床应用价值，为ENB技术更好的发展提供帮助。

## 1 材料与方法

### 1.1 设备与材料

① 电磁导航支气管镜系统（美国Super Dimension公司），包含电磁定位板、导航定位装置、延伸的工作通道（extended working channel, EWC）、计算机软件及显示器。②电视胸腔镜系统（德国KARL STORZ公司）。

### 1.2 临床资料

回顾性分析2017年10月-2018年10月于青岛大学附属医院胸外科接受ENB矢量定位后进行VATS肺切除术的22例患者资料。其中男性8例，女性14例，平均年龄59.9岁（范围36岁-80岁）。所有患者术前胸部CT提示只有1个肺小结节，且结节距离肺脏层胸膜均 < 3.5 cm，可行胸腔镜下肺楔形切除术。记录内容包括肺小结节的直径、位置、距离脏层胸膜的距离、定位成功率、中转开胸率、胸腔镜下肺楔形切除时间、术中情况、术后并发症等。

### 1.3 ENB矢量定位步骤和手术方法

① 将患者术前胸部CT三维图像传输至ENB系统，建立相应的虚拟支气管树，在模拟图像中对可疑病灶的位置及大小进行标记。确认规划路径后，将患者置于仰卧位，于单腔气管插管全身麻醉下，通过EWC置入LG完成注册后撤出纤维支气管镜和LG；②将单腔气管导管更换为双腔支气管导管，在便携式纤维支气管镜和实时导航监测下，按照规划路径将LG推进到计划位置，此时记录LG探头与病灶之间的方向和距离，在确定两者相对位置后，将LG固定在相应支气管入口处，撤出便携式纤维支气管镜（图 1A）；③将患者置于健侧卧位，此过程中注意保持LG位置固定，然后实行单肺通气，采用胸腔镜进行手术操作，由助手轻推LG，使脏层胸膜表面形成“帐篷”样突起，使用电刀标记（图 1B）。移除LG后，由于术侧肺塌陷以及LG对肺组织的刚性作用，术者能够准确地进行病灶及周围肺组织楔形切除术。移除标本，进行快速冰冻病理检查，根据冰冻病理结果和患者情况，决定是否继续进行腔镜下解剖性肺切除术加系统性淋巴结清扫术。

**1:**
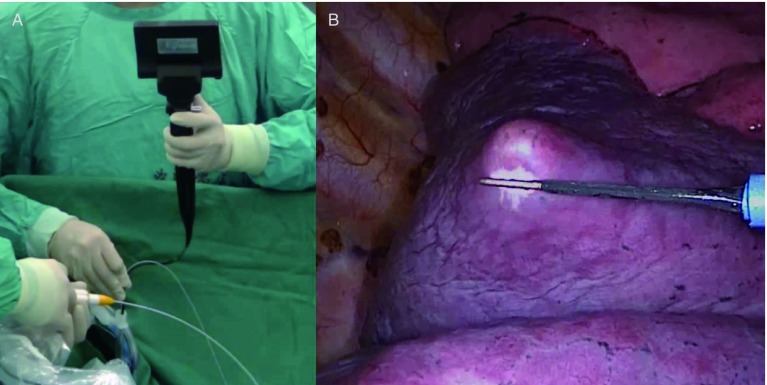
手术图片。A：经双腔支气管导管插入导航定位装置和便携式支气管镜；B：由导航定位装置的探头在脏层胸膜上顶起的“帐篷”样突起，使用电刀烧灼作为标记。 Operation pictures. A: The locatable guide and portable bronchoscope were inserted through the double-lumen endobronchial tube; B: The protuberant tent-like visceral pleura propped by the locatable guide probe with cauterized by electrotome.

## 2 结果

22例患者的22个孤立性结节（5 mm-20 mm）在ENB引导下矢量定位成功率100.0%，其中结节位于右肺上叶1例，右肺中叶2例，右肺下叶9例，左肺上叶3例，左肺下叶7例；结节大小（11.0±3.6）mm；在影像学上表现为GGO 18例，实性结节2例，混合磨玻璃结节2例。病灶距离胸膜垂直距离平均（16.5±6.2）mm，VATS楔形切除成功率100.0%，无中转开胸。无定位点LG发生移位（0.0%），无定位相关并发症（0.0%）。ENB系统显示屏LG与病灶距离为（14.5±10.1）mm，离体标本标记与病灶距离为（15.3±11.0）mm，矢量定位法操作时间（包括更换气管插管时间）为（17.5±4.2）min。术后患者平均住院时间（3.8±1.2）d，无手术后死亡患者（0.0%）。所有患者术后石蜡切片病理结果：原位腺癌（adenocarcinoma *in situ*, AIS）（3例，13.6%），微浸润性腺癌（microinvasive adenocarcinoma, MIA）（14例，63.7%），浸润性腺癌（invasive adenocarcinoma, IA）（2例，9.1%），慢性炎症（chronic inflammatory）（3例，13.6%）（表 1）。

**1:** 临床概况 Clinical profiles

Variables	Data
Gender	22
Male	8
Female	14
Average age (yr)	59.9
Nodule type	
Ground glass	18
Solid	2
Mixed	2
Nodule location	
Left upper lobe	3
Left lower lobe	7
Right upper lobe	1
Right middle lobe	2
Right lower lobe	9
Nodule size (Mean±SD, mm)	11.0±3.6
Estimated distance from the visceral pleura (Mean±SD, mm)	16.5±6.2
Final pathology for resected tissue	
AIH	3
MIA	14
IA	2
Chronic inflammatory	3
Distance between the LG probe and lesion on screen (Mean±SD, mm)	14.5±10.1
Distance between the lesion and mark on the dissected specimen (Mean±SD, mm)	15.3±11.0
The localization time (Mean±SD, min)	17.5±4.2
Length of hospital stay (Mean±SD, d)	3.8±1.2
AIH: adenocarcinoma *in situ*; MIA: microinvasive adenocarcinoma; IA: invasive adenocarcinoma; LG: locatable guide probe. The localization time includes the time of changing the double lumen tube.	

## 3 讨论

对于一些直径 < 1 cm的结节和一些质地与周围正常肺组织难以区分的GGO而言，精确定位是胸外科临床医师面临的重要问题，而胸腔镜手术技术在一定程度上限制了手术医师对病灶的直接触摸，导致一些腔镜手术不得不中转开胸，更有极少数病例即使行开胸手术后在准确寻找病灶上也极为困难^[14]^。除了CT引导的穿刺针定位以外，ENB引导的肺结节染料定位法是近几年较为常用的术前结节定位的方法之一^[15, 16]^。ENB不仅可以到达常规纤维支气管镜无法到达的肺周围性病变的位置，并且该技术无射线辐射、使用方便、无需使用造影剂、无需破坏胸膜，相比CT引导的穿刺定位有着较低的并发症发生率^[10, 15]^。但是，由于肺实质的复杂结构以及ENB染料定位后与外科手术之间的时间间隔，可能会出现染料向周围肺组织扩散，导致染色区域变大从而影响准确定位^[17, 18]^。为了更准确地对肺结节进行定位，联合应用CT引导的hook wire穿刺定位及ENB引导的亚甲兰染色定位的技术也被报道。陈海泉等^[19]^曾报道联合应用亚甲兰及hook wire对右肺上叶直径0.6 cm的结节进行成功定位，但是该过程需要CT扫描来确认hook wire的位置，增加了患者和术者的射线辐射风险^[20]^。

我们采用的矢量定位法通过以LG探头为定位标志，而非使用染料或者定位钩，在ENB系统的虚拟支气管树和三维CT视图中到达病灶并计算两者距离和相对位置，在肺脏层胸膜上形成“帐篷”样的突起，避免了上述方法的不利。Marino等^[17]^利用亚甲兰定位成功率达到了97.2%，而我们的定位成功率达到了100.0%，且22例病灶均获得了完整的腔镜下楔形切除，术中无中转开胸，无其他术中并发症，证实了该方法的有效性及安全性。同时，该过程没有采用术中透视或者CT扫描，而是在电视胸腔镜直视下完成对病灶的定位与标记，避免了患者和术者的额外射线辐射，简化了相应的手术流程。

虽然该方法有一定的优势，但同时也存在一定的不足。ENB系统在国内仍是一项新技术，仍未在国内广大医院广泛应用。另外，该技术对术者的解剖知识及操作水平的要求比较高，因此该方法需要术者经过系统性的培训和不断的学习来积累一定的经验^[21]^。除此以外，患者的合理筛选对于定位操作也尤为重要，我们的经验提示该方法适用于外1/3的外周结节。通过我们对上述病例的实践总结，有两点关键的因素影响定位的精确度：首先是LG探头的位置应尽可能靠近脏层胸膜，使得“帐篷”更加显而易见和稳定；其次是将患者由平卧位转至健侧卧位时，要特别注意轻柔搬动以防止LG脱落。当然，X线透视或者便携CT也是确定LG位置的方法，但随之而来的额外辐射增加的风险不可忽视。

总之，我们的研究经验证明了ENB矢量定位法的安全性和准确性。该方法不仅能够做到对肺外周结节精准定位，而且能减少特殊材料的使用；操作上对胸膜的创伤小，也避免了增加定位操作时间。虽然仍存在一定的局限性，但可以为肺小结节诊断性切除手术的术中准确定位提供一种选择。胸外科医师可以进一步研究该方法并应用到临床操作中。
